# Research Progress and Challenges in Targeting GLUT1 in Tumors

**DOI:** 10.3390/ijms27209225

**Published:** 2026-10-17

**Authors:** Taohua Chen, Bi Peng, Xinya Yuan, Yizhou Liu, Jianing Zhang, Yizhen Su, Guangyuan Hu, Zhen Tao, Guoxian Long

**Affiliations:** Department of Oncology, Tongji Hospital, Tongji Medical College, Huazhong University of Science and Technology, Wuhan 430030, China

**Keywords:** *SLC2A1*, GLUT1, aerobic glycolysis, heterogeneity, metabolic–immune crosstalk, therapeutic window

## Abstract

The solute carrier family 2 member 1 (*SLC2A1*), which encodes glucose transporter 1 (GLUT1), has long been recognized as a key driver of aerobic glycolysis in cancer. However, accumulating evidence has substantially expanded this view, revealing that GLUT1 functions not only as a critical mediator of metabolic reprogramming but also as an important regulator linking tumor metabolism to immune evasion. Notably, GLUT1 expression, regulation, and functional dependence are highly context-dependent across tumor types, disease states, and cellular compartments. Recent studies further link GLUT1-dependent glycolytic flux to immune regulation through glucose competition, lactate accumulation, and metabolite-dependent epigenetic programs within the tumor microenvironment. Reflecting this evolving understanding, therapeutic strategies targeting GLUT1 have progressed from simple metabolic inhibition to approaches that exploit tumor-specific metabolic dependencies and extend to regulated cell death, combination therapy, precision delivery, and selective modulation of malignant and non-malignant cellular compartments. These advances support a shift from expression-centered targeting toward strategies guided by functional GLUT1 dependence and cellular context, while recognizing the metabolic and physiological constraints that shape the therapeutic window. In this review, we systematically summarize recent advances in GLUT1 expression heterogeneity, multilayered regulatory networks, metabolic–immune crosstalk, diagnostic and prognostic relevance, and therapeutic targeting, and discuss how these insights may refine biomarker development, patient selection, and the clinical translation of GLUT1-directed precision metabolic therapy.

## 1. Introduction

Aerobic glycolysis, also known as the Warburg effect, has long been considered a defining metabolic hallmark of malignant cells, in which glucose is preferentially metabolized via glycolysis rather than oxidative phosphorylation despite adequate oxygen availability. However, accumulating evidence has challenged this oversimplified view, demonstrating that glycolysis and oxidative phosphorylation frequently coexist and cooperate within tumor cells to support their metabolic demands [1,2]. Nevertheless, targeting aerobic glycolysis remains a promising therapeutic strategy, as disruption of glucose utilization can impair tumor cell proliferation, migration, and invasion [3,4,5] and enhance responses to radiotherapy, chemotherapy, targeted therapy, and immunotherapy [6,7,8,9,10]. Within this metabolic framework, glucose transporter 1 (GLUT1), encoded by solute carrier family 2 member 1 (*SLC2A1*), represents a critical determinant of glucose availability and glycolytic activity. GLUT1 is frequently upregulated across diverse solid tumors and hematological malignancies, supporting enhanced glucose uptake and metabolic adaptation in malignant cells [11,12,13,14,15,16,17,18,19,20,21,22,23]. Consequently, GLUT1 has emerged as a candidate target for metabolism-directed cancer therapy, with increasing interest in its broader roles beyond tumor-cell metabolism.

Glucose transport across the plasma membrane can represent an important rate-limiting step in glycolytic metabolism. GLUT1, encoded by *SLC2A1*, is a major transporter responsible for basal glucose uptake and is broadly expressed in several normal tissues, including erythrocytes and brain microvascular endothelial cells at the blood–brain barrier (BBB) of the central nervous system (CNS). During early development, GLUT1 deficiency depletes cellular energy and restricts endothelial proliferation, impairing angiogenesis and CNS homeostasis [24,25]. In adulthood, however, the consequences of GLUT1 loss appear to be highly cell- and context-dependent. Conditional deletion of GLUT1 in adult astrocytes can induce compensatory metabolic reprogramming that preserves, or in some settings even enhances, glucose utilization and neuronal support [26,27]. Similarly, GLUT1 deficiency does not impair human erythroblast proliferation, differentiation, or enucleation, suggesting that terminal erythroid differentiation can remain relatively preserved despite substantial GLUT1 loss [28]. Importantly, however, these compensatory responses should not be extrapolated to suggest that systemic GLUT1 inhibition is broadly tolerated. GLUT1 has a nonredundant role in transporting circulating glucose across the BBB, and *SLC2A1* haploinsufficiency causes GLUT1 deficiency syndrome (Glut1DS, also known as De Vivo syndrome), in which impaired cerebral glucose availability produces a spectrum of neurological manifestations, including seizures, developmental impairment, and movement disorders [29,30,31]. Human genetic evidence has directly linked Glut1DS to haploinsufficiency of the BBB glucose transporter, while experimental models further demonstrate that reduced GLUT1 disrupts cerebral microvascular development and brain glucose homeostasis [24,25,29,32]. These observations identify cerebral glucose transport as an important physiological constraint on systemic GLUT1 inhibition and indicate that any therapeutically exploitable window is likely to depend on inhibitor selectivity, the magnitude and duration of target inhibition, systemic exposure, and tissue distribution.

Beyond these safety considerations, the clinical translation of GLUT1-directed therapies remains constrained by several additional biological challenges. GLUT1 expression and activity are strongly influenced by tumor lineage, histological subtype, and differentiation state, resulting in substantial intertumoral and intratumoral heterogeneity that limits the feasibility of universal targeting strategies. Moreover, GLUT1-dependent glycolytic metabolism is not a fixed metabolic state but a dynamic phenotype shaped by microenvironmental stress and interactions with stromal and immune cells, and multilayered oncogenic and tumor-suppressive regulation. The inherent metabolic plasticity of cancer cells can enable compensatory metabolic pathways following GLUT1 inhibition, thereby attenuating therapeutic responses. In addition, the lack of reliable biomarkers for patient selection continues to complicate the identification of tumors that are functionally dependent on GLUT1 rather than merely expressing high levels of the transporter. These considerations indicate that the translational potential of GLUT1 cannot be inferred from transporter expression or inhibitor potency alone. Rather, it depends on the biological context in which GLUT1 constitutes a functional metabolic dependency and on whether therapeutically relevant target inhibition can be achieved without unacceptable effects on physiologically GLUT1-dependent tissues. In this review, we therefore discuss the heterogeneous expression and multilayered regulation of GLUT1, its diagnostic and prognostic relevance, and emerging therapeutic strategies targeting GLUT1 in both malignant cells and non-malignant components of the tumor microenvironment (TME), while also considering emerging links between GLUT1-dependent metabolism and epigenetic regulation and the biological constraints that shape its therapeutic window and clinical translation.

## 2. Heterogeneous Expression of GLUT1 in Tumors and Its Prognostic Implications

Current evidence indicates that GLUT1 is highly expressed across a broad spectrum of solid malignancies and is frequently associated with adverse clinical outcomes [11,12,13,14,15,16,17,18,19,20,21,22,23]. However, accumulating data suggest that the expression patterns and biological significance of GLUT1 are highly context-dependent rather than uniform across tumor types.

The heterogeneity of GLUT1 biology extends beyond differences among tumor types, encompassing pathological subtypes, disease stages, and spatially distinct regions within individual tumors. For example, in lung cancer, GLUT1 expression differs markedly between histological subtypes. Expression is higher in squamous cell carcinoma than in adenocarcinoma, yet its prognostic significance is observed only in adenocarcinoma [33]. Among lung neuroendocrine tumors, GLUT1 is minimally expressed in typical and atypical carcinoids but is significantly upregulated in aggressive large-cell neuroendocrine and small-cell carcinomas [12]. Thyroid cancers display a gradient of expression, with levels increasing in parallel with dedifferentiation; undifferentiated thyroid carcinomas exhibit the highest GLUT1 expression [14]. Expression dynamics can also shift during disease progression: in breast carcinoma, in situ lesions show higher GLUT1 levels than invasive tumors, suggesting that tumor dependence on GLUT1 may decrease as malignancy advances [34]. Moreover, metabolic specialization exists within the same tumor: in collectively invading lung cancer cells, leading cells preferentially rely on mitochondrial respiration, whereas trailing cells depend on GLUT1-mediated aerobic glycolysis, highlighting intratumoral metabolic heterogeneity [35]. Conflicting findings further underscore the complexity of GLUT1 biology. In gastric cancer, reported *SLC2A1* expression ranges from low and non-prognostic to high and strongly associated with tumor progression [17,36]. In colorectal cancer, GLUT1 expression is similarly significantly upregulated [18]. Yet, its association with prognosis is contradictory. Some studies suggest no correlation with prognosis [37], while another study indicates that high GLUT1 expression actually predicts a favorable postoperative prognosis in patients with liver metastases from colorectal cancer [38].

Although adaptive engagement of alternative glucose transporters, particularly GLUT3, can preserve glucose uptake under metabolic stress and thereby uncouple GLUT1 abundance from functional glucose dependence [21,37], this mechanism alone is unlikely to account for the divergent findings observed across tumor contexts. These observations suggest that the biological and prognostic significance of GLUT1 is shaped not only by its abundance but also by the regulatory context that determines its expression and activity, thereby providing a rationale for examining the multilayered mechanisms governing GLUT1 abundance and function in tumors.

## 3. Regulatory Networks of GLUT1 (*SLC2A1*) in Tumor Tissues

This context-dependent heterogeneity in GLUT1 expression reflects the multilayered regulation of *SLC2A1*, which is controlled by coordinated mechanisms operating at the transcriptional, post-transcriptional, and post-translational levels. Recent studies have begun to delineate these regulatory networks, revealing how diverse molecular inputs collectively shape GLUT1 abundance across tumor contexts.

**Figure 1:**
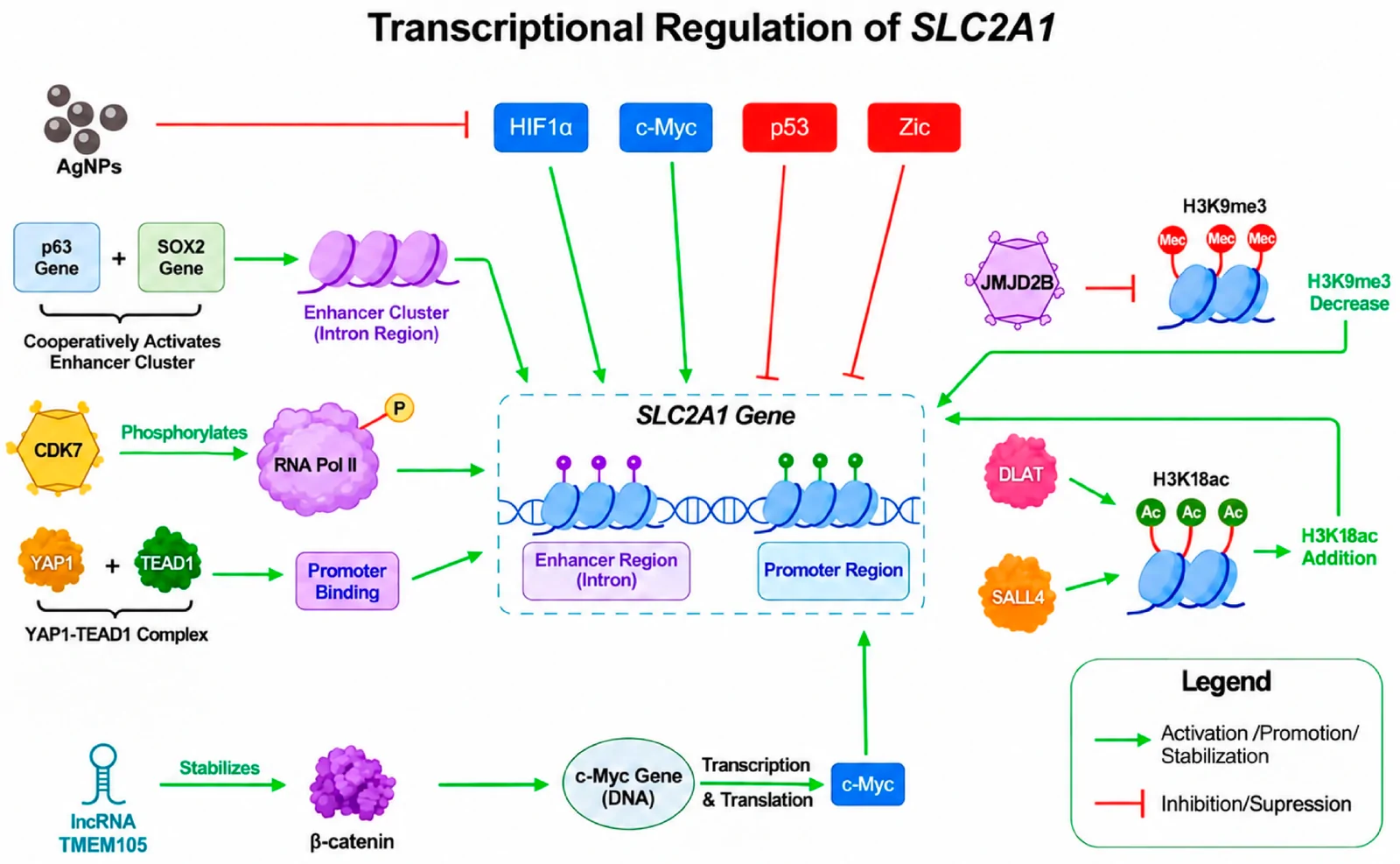
Transcriptional regulation of *SLC2A1*. Abbreviations: *SLC2A1*, solute carrier family 2 member 1; AgNPs, silver nanoparticles; HIF-1α, hypoxia-inducible factor 1-alpha; SOX2, sex-determining region Y-box 2; CDK7, cyclin-dependent kinase 7; RNA Pol II, RNA polymerase II; YAP1, Yes-associated protein 1; TEAD1, TEA domain transcription factor 1; Zic, zinc finger of the cerebellum proteins; JMJD2B, Jumonji domain-containing protein 2B; H3K9me3, histone H3 lysine 9 trimethylation; DLAT, dihydrolipoyl transacetylase; SALL4, spalt-like transcription factor 4; H3K18ac, histone H3 lysine 18 acetylation; lncRNA, long non-coding RNA.

At the transcriptional level, hypoxia-inducible factor-1alpha (HIF-1α), p53, and c-Myc (encoded by the well-known *MYC* oncogene) are well-established regulators of glycolytic reprogramming and represent classical transcriptional determinants of *SLC2A1* expression [39,40,41,42,43]. Recent studies have considerably expanded this regulatory landscape. Evidence from breast cancer models has shown that silver nanoparticles (AgNPs) attenuate hypoxia-induced HIF-1α protein accumulation and hypoxia-responsive element (HRE)-dependent transcriptional activity, thereby reducing induction of the downstream HIF target gene *SLC2A1* [44]. In squamous cell carcinoma, the transcription factor p63 cooperates with sex-determining region Y-box 2 (SOX2) to activate an intronic enhancer cluster within the *SLC2A1* locus, thereby driving gene transcription [45]. In breast cancer, the transcriptional co-activator Yes-associated protein 1 (YAP1) forms a transcriptional complex with TEA domain transcription factor 1 (TEAD1), which directly engages the *SLC2A1* promoter to enhance its expression [46]. Similarly, in lung cancer, cyclin-dependent kinase 7 (CDK7) enhances *SLC2A1* transcription through phosphorylation of RNA polymerase II [47]. In contrast, members of the zinc finger of the cerebellum proteins (Zic) family act as transcriptional repressors in colorectal cancer by directly binding to the *SLC2A1* promoter and suppressing its activity [48]. Epigenetic regulation further adds complexity to the transcriptional control of *SLC2A1*. In colorectal cancer cells, Jumonji domain-containing protein 2B (JMJD2B) relieves transcriptional repression by reducing histone H3 lysine 9 trimethylation (H3K9me3) at the *SLC2A1* promoter [49]. In hepatocellular carcinoma, both dihydrolipoyl transacetylase (DLAT) and spalt-like transcription factor 4 (SALL4) have been shown to enhance *SLC2A1* transcription through increased histone H3 lysine 18 acetylation (H3K18ac) within the promoter region [50,51]. In addition, long non-coding RNAs (lncRNAs) contribute to the fine regulation of *SLC2A1* transcription. In pancreatic cancer, lncRNA TMEM105 stabilizes β-catenin through a proteasome-dependent mechanism, leading to c-Myc upregulation and subsequent activation of *SLC2A1* transcription [43] (Figure 1).

**Figure 2:**
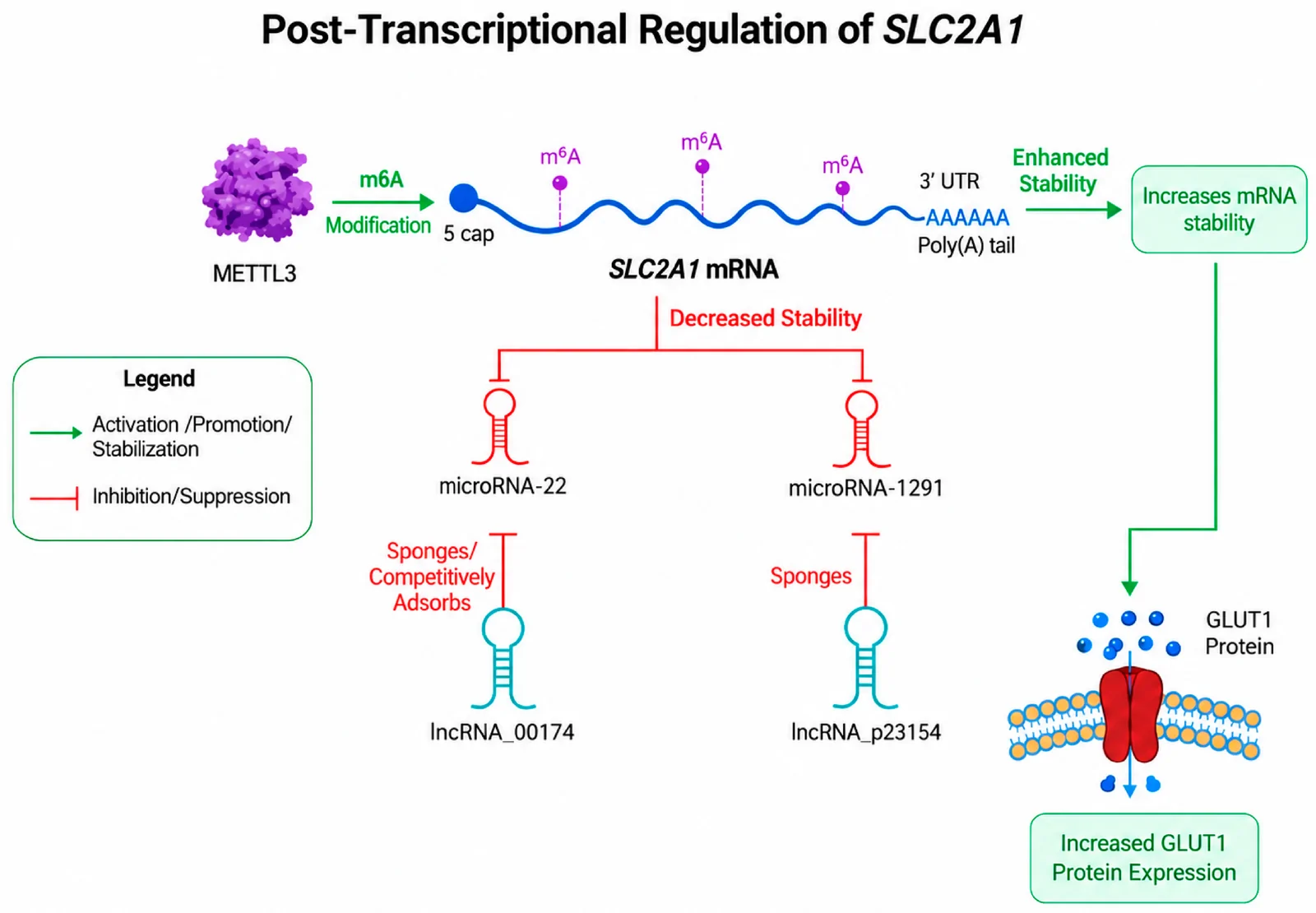
Post-transcriptional regulation of *SLC2A1*. Abbreviations: *SLC2A1*, solute carrier family 2 member 1; GLUT1, glucose transporter 1; METTL3, methyltransferase-like 3; m6A, N6-methyladenosine; miR, microRNA; lncRNA, long non-coding RNA; 3′ UTR, 3′ untranslated region; poly(A), polyadenylate tail.

Post-transcriptional regulation of *SLC2A1* is primarily mediated through mechanisms that govern mRNA stability. On one hand, methyltransferase-like 3 (METTL3), which is frequently upregulated in colorectal cancer, enhances the stability of *SLC2A1* mRNA through N6-methyladenosine (m6A) modification, thereby promoting its expression [52,53]. On the other hand, several microRNAs, including miR-22 and miR-1291, which are downregulated in breast cancer and renal cell carcinoma, respectively, directly target the 3′ untranslated region (3′ UTR) of *SLC2A1* mRNA and accelerate its degradation [54,55]. In addition to direct miRNA-mediated regulation, an emerging layer of post-transcriptional control involves non-coding RNAs functioning as competing endogenous RNAs (ceRNAs). Long non-coding RNAs, including lncRNA-00174 and lncRNA-p23154, act as molecular sponges that sequester specific miRNAs, thereby attenuating miRNA-mediated silencing and stabilizing *SLC2A1* transcripts in glioma and oral squamous cell carcinoma [56,57] (Figure 2).

**Figure 3:**
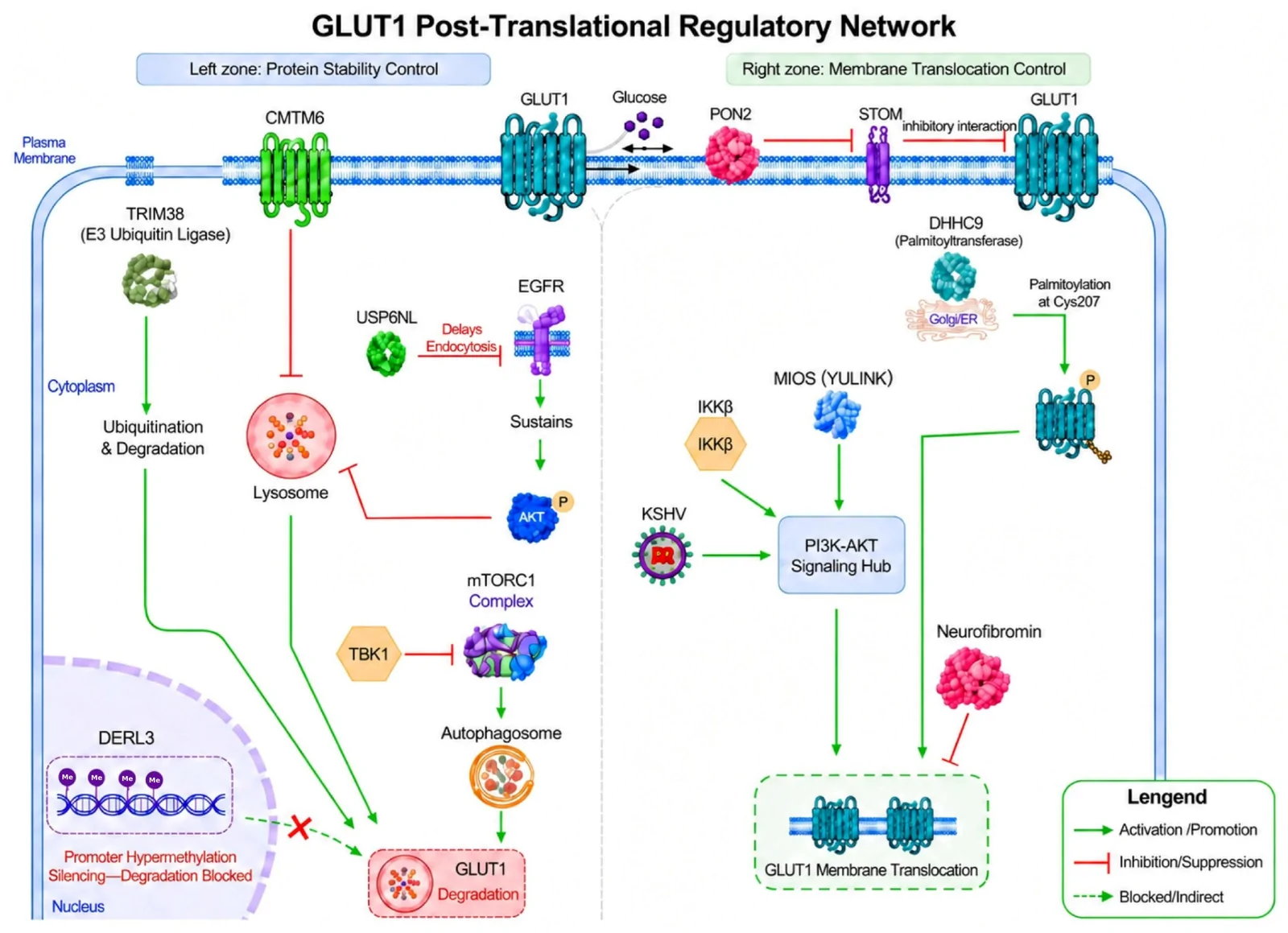
Post-translational regulation of GLUT1. Abbreviations: GLUT1, glucose transporter 1; TRIM38, tripartite motif-containing protein 38; CMTM6, CKLF-like MARVEL transmembrane domain-containing 6; *DERL3*, derlin-3; USP6NL, ubiquitin-specific protease 6 N-terminal-like protein; EGFR, epidermal growth factor receptor; AKT, serine/threonine kinase AKT; TBK1, TANK-binding kinase 1; mTORC1, mechanistic target of rapamycin complex 1; PI3K, phosphatidylinositol 3-kinase; KSHV, Kaposi sarcoma-associated herpesvirus; IKKβ, inhibitor of nuclear factor kappa-B kinase subunit beta; MIOS, meiosis regulator for oocyte development, also termed YULINK; PON2, paraoxonase 2; STOM, stomatin; DHHC9, zinc finger DHHC-type palmitoyltransferase 9; Cys207, cysteine residue 207.

Compared with the relatively limited understanding of translational regulation, the post-translational regulation of GLUT1 has been more extensively characterized and serves as a critical determinant of its functional activity. Current evidence indicates that post-translational regulation of GLUT1 is primarily mediated through two interconnected mechanisms: the control of protein stability and the regulation of membrane trafficking. The maintenance of GLUT1 protein stability involves both the ubiquitin–proteasome system and the autophagy–lysosomal pathway. In bladder cancer, tripartite motif-containing protein 38 (TRIM38) promotes GLUT1 ubiquitination and subsequent degradation [58]. In colorectal cancer, CKLF-like MARVEL transmembrane domain-containing 6 (CMTM6) inhibits lysosome-mediated GLUT1 degradation, whereas promoter-hypermethylation-induced silencing of derlin-3 (*DERL3*) compromises the degradation of its downstream target GLUT1 [59]. Similarly, in breast cancer, ubiquitin-specific protease 6 N-terminal-like protein (USP6NL) delays epidermal growth factor receptor (EGFR) endocytosis, thereby sustaining serine/threonine kinase (AKT) activation and preventing lysosomal degradation of GLUT1 [60]. In addition, TANK-binding kinase 1 (TBK1) suppresses mechanistic target of rapamycin complex 1 (mTORC1)-dependent autophagy, leading to reduced GLUT1 degradation and enhanced membrane localization in colorectal cancer cells [61]. Regulation of GLUT1 membrane trafficking constitutes another major layer of post-translational control. In glioblastoma, the zinc finger DHHC-type palmitoyltransferase 9 (DHHC9) catalyzes palmitoylation of GLUT1 at Cys207, thereby promoting its translocation to the plasma membrane [62]. Notably, the phosphatidylinositol-3-kinase (PI3K)/AKT signaling pathway functions as a central regulatory hub for GLUT1 membrane trafficking by integrating multiple upstream signals. Meiosis regulator for oocyte development (MIOS; also termed YULINK), Kaposi sarcoma-associated herpesvirus (KSHV), and inhibitor of nuclear factor kappa-B kinase subunit beta (IKKβ) have all been shown to promote GLUT1 membrane translocation through activation of the PI3K/AKT pathway [63,64,65]. Conversely, neurofibromin exerts an inhibitory effect on GLUT1 trafficking [66]. Furthermore, paraoxonase 2 (PON2) enhances glucose transport in pancreatic cancer cells by modulating the inhibitory interaction between GLUT1 and stomatin (STOM) [67] (Figure 3).

Regulatory mechanisms operating at different molecular levels do not function independently; rather, they are integrated into highly interconnected regulatory networks. Pancreatic cancer provides a representative example of such coordinated regulation. Forkhead box D1 (FOXD1) simultaneously activates the transcription of both *SLC2A1* and lncRNA HOXA11-AS. In turn, lncRNA HOXA11-AS functions as a ceRNA by sequestering miR-148b-3p, thereby preventing RNA-induced silencing complex (RISC)-mediated degradation of *SLC2A1* mRNA [68]. This regulatory circuit illustrates how transcriptional and post-transcriptional mechanisms can act in concert to sustain *SLC2A1* expression. Although individual regulators of *SLC2A1* are increasingly well characterized, their relative contributions and temporal hierarchy across tumor states remain incompletely resolved. Most studies examine individual regulatory nodes under defined experimental conditions, limiting our ability to define context-specific combinations of regulators and their temporal dynamics. Resolving these relationships will be important for distinguishing tumor states in which *SLC2A1* expression reflects a stable metabolic dependency from those in which it represents a transient adaptive response.

## 4. GLUT1-Dependent Glycolysis at the Metabolic–Immune Interface

The regulatory networks described above determine not only GLUT1 abundance and membrane availability but also the capacity of individual cellular compartments to sustain glucose uptake and glycolytic flux. Once established, this metabolic state can extend beyond cell-autonomous bioenergetics because malignant and non-malignant cells draw on a shared pool of nutrients and exchange metabolites within the TME. Current mechanistic studies have most directly linked GLUT1-associated glycolysis to immune regulation through two interconnected consequences: reduced extracellular glucose availability and increased lactate production. These mechanisms are unlikely to encompass the full spectrum of metabolic effects downstream of GLUT1, but other routes remain less well resolved in terms of their direct dependence on GLUT1 and are therefore not considered in detail here.

One well-defined interaction originates within malignant cells. Increased tumor-cell glycolysis can reduce glucose availability to tumor-infiltrating lymphocytes and thereby constrain the metabolic program required for antitumor effector function. In an antigenic murine sarcoma model, forced expression of GLUT1 in tumor cells increased glycolytic activity and shifted glucose acquisition toward the malignant compartment, reducing relative glucose uptake by tumor-infiltrating lymphocytes; more broadly, tumor-imposed glucose restriction suppressed T cell mTOR activity, glycolytic capacity, and IFN-γ production [69]. A more direct GLUT1-dependent mechanism has subsequently been demonstrated in hepatocellular carcinoma, where tumor-cell ubiquitin-specific protease 14 (USP14) deubiquitinates and stabilizes GLUT1, enabling malignant cells to outcompete CD8^+^ T cells for glucose and thereby impair their cytotoxic function [70].

GLUT1-dependent immune regulation, however, is not restricted to tumor-cell glucose consumption. GLUT1 activity within non-malignant compartments can itself shape the local immune state. In non-small-cell lung cancer (NSCLC), macrophage-specific deletion of *SLC2A1* increased the abundance, spatial homogeneity, and effector function of intratumoral CD8^+^ T cells, whereas tumor-cell-specific *SLC2A1* depletion did not reproduce this phenotype. Notably, loss of macrophage *SLC2A1* did not increase glucose uptake by CD8^+^ T cells, indicating that the immunological effect in this setting could not be explained by simple redistribution of extracellular glucose [71]. These observations highlight the compartment-specific nature of GLUT1-dependent immune regulation: metabolic programs initiated in malignant and non-malignant cells can converge on antitumor immunity through mechanistically distinct routes.

Whereas glucose competition operates through depletion of a shared nutrient pool, lactate provides a metabolite-mediated route through which glycolytic activity can be communicated between cellular compartments. In melanoma models, lactate dehydrogenase A (LDHA)-dependent lactic acid production by tumor cells suppresses nuclear factor of activated T cells (NFAT) activation and IFN-γ production of T and natural killer (NK) cells, whereas tumor-infiltrating regulatory T cells can import and utilize lactate to maintain suppressive function under glucose-restricted conditions [72,73]. These studies did not establish GLUT1 as the specific upstream determinant of lactate production, but they define the immunological consequences of a major metabolite generated by highly glycolytic tumors. Lactate can therefore influence immune function not only through its metabolic effects but also by serving as a substrate for regulatory modifications that alter gene expression.

The discovery of histone lysine lactylation provided a mechanistic basis for this latter effect. Glycolysis-derived lactate can contribute to histone lactylation and thereby couple cellular metabolic state to chromatin-dependent transcription [74]. Subsequent tumor studies have placed GLUT1 directly upstream of this metabolic–epigenetic process in distinct cellular settings. In glioblastoma, tumor-derived signals activate the protein kinase R-like endoplasmic reticulum kinase (PERK)/transcription factor 4 (ATF4) signaling in monocyte-derived macrophages, inducing macrophage-intrinsic GLUT1 expression and glycolysis. In this setting, glucose metabolized within the macrophage generates intracellular lactate that contributes to histone lactylation at the IL-10 locus; suppression of glycolysis or lactate production reduces IL-10 expression and weakens macrophage-mediated inhibition of T cell activity [75]. Isotope-tracing experiments further showed that glucose-derived carbon contributed more substantially to histone lactylation than extracellular lactate in this model. Thus, although the initiating signal originates from the tumor, the GLUT1-dependent metabolic and epigenetic program is executed within the myeloid cell and subsequently alters T cell function.

A distinct configuration has been identified in hepatocellular carcinoma. Angiopoietin-like 4 (ANGPTL4) derived from tumor-associated fibroblasts (CAFs) interacts with GLUT1 on malignant cells and increases glucose-transport activity without increasing transporter abundance or membrane localization. The resulting increase in tumor-cell glucose utilization and lactate production enhances histone H3 lysine 18 lactylation (H3K18la) at the programmed cell death 1 ligand 1 (*PD-L1*) promoter and increases *PD-L1* expression; GLUT1 depletion attenuates both H3K18la and *PD-L1* induction and restores CD8^+^ T cell activity [76]. Together with the glioblastoma model, these findings show that GLUT1-linked lactylation can support immunosuppressive transcriptional programs in different cellular compartments: within immune cells through cell-intrinsic metabolic reprogramming, or within malignant cells in response to microenvironmental signals.

Lactate-dependent regulation can extend beyond histone modification alone. In tumor-infiltrating myeloid cells, lactate accumulated within the TME induces H3K18la at the *METTL3* locus and increases *METTL3* expression; lactylation of METTL3 itself further enhances m6A-dependent regulation of Janus kinase 1 (JAK1) translation and signal transducer and activator of transcription 3 (STAT3) activation, reinforcing an immunosuppressive myeloid state [77]. Because the upstream source of lactate was not assigned specifically to GLUT1 in that study, this pathway is better regarded as a lactate-responsive regulatory module operating within a glycolytic TME rather than as a direct GLUT1/METTL3 axis. Nevertheless, it demonstrates how a glycolytic metabolite can propagate its regulatory effects from chromatin modification to epitranscriptomic control.

More recent evidence indicates that metabolic–epigenetic coupling can, in selected tumor contexts, feed back onto the glycolytic program itself. In hepatocellular carcinoma, glycolysis-associated H3K18la increases chromatin accessibility at the transcriptional repressor GATA binding 1 (*TRPS1*) and ETS variant transcription factor 1 (*ETV1*) promoters. TRPS1 subsequently reinforces GLUT1 expression and glycolytic activity, whereas ETV1 promotes C-C motif chemokine ligand 2 (CCL2)-dependent exclusion of CD8^+^ T cells [78]. This configuration establishes a feed-forward relationship in which a metabolite-derived chromatin modification can reinforce both glycolytic metabolism and an immune-evasive transcriptional state. Such findings extend histone lactylation beyond a passive marker of glycolytic activity and identify it, at least in defined tumor contexts, as a regulatory component capable of maintaining metabolic and immune phenotypes.

Taken together, current evidence supports a context-dependent coupling between GLUT1-associated glucose metabolism and tumor immune regulation. Increased glucose flux can influence neighboring cells through competition for glucose and production of lactate, while lactate-dependent epigenetic regulation can convert metabolic changes into more persistent immunoregulatory transcriptional programs. In selected tumor settings, these programs further reinforce glycolytic activity or immune exclusion. The specific organization of this network varies according to the GLUT1-dependent cellular compartment, the source and handling of lactate, the epigenetic targets engaged, and the availability of compensatory metabolic pathways. Such heterogeneity does not argue against the existence of a metabolic–immune circuit; rather, it determines how the circuit is wired and which nodes constitute therapeutically tractable dependencies.

## 5. Diagnostic Significance of GLUT1 in Tumors

Because total GLUT1 expression does not necessarily reflect transporter activity or functional metabolic dependence, its interpretation as a clinical biomarker remains context-dependent. The clinical utility of GLUT1 in oncology is expanding beyond conventional immunohistochemical characterization toward molecular imaging, prognostic stratification, and recurrence risk prediction. However, GLUT1 expression alone has limited diagnostic specificity. GLUT1 positivity can also be detected in certain benign lesions, restricting its use as a standalone diagnostic marker [79,80,81]. Nevertheless, GLUT1 immunoreactivity can provide valuable adjunctive information in the evaluation of suspicious lesions, including squamous lesions of the head and neck [82]. Conversely, the absence of GLUT1 expression does not exclude malignancy, as exemplified by the downregulation of GLUT1 in approximately 55% of malignant melanomas [83]. Accordingly, integrating GLUT1 with additional biomarkers or complementary detection approaches may improve diagnostic discrimination. For instance, combined analysis of GLUT1 and other cell-surface markers, including prion protein, has shown potential for identifying high-risk colorectal adenomas and early colorectal cancer progression [84].

Beyond diagnosis, GLUT1-based signatures have shown promise for early detection and prognostic assessment. A combined *SLC2A1*/*MPST* signature exhibits strong diagnostic performance in endometrial cancer [85], while a three-protein panel including GLUT1 can stratify patients with stage I-IIA lung adenocarcinoma according to recurrence risk and survival outcomes [86]. In breast cancer, the *SLC2A1* XbaI G>T single-nucleotide polymorphism has been associated with tumor grade and proliferative capacity, suggesting potential prognostic relevance [87].

Molecular imaging represents another emerging application of GLUT1-based detection. In fluorescence molecular endoscopy, GLUT1 has been evaluated as an imaging target for esophageal squamous cell carcinoma and high-grade intraepithelial neoplasia. Topical administration of the fluorescent tracer 2-DG 800CW enabled discrimination of high-grade lesions from low-grade lesions and normal tissue with a sensitivity of 80% and a specificity of 83% [23]. Similarly, the GLUT1-targeted tracer WZB117-CY7.5 selectively binds cell-surface GLUT1 and has been successfully applied in near-infrared fluorescence image-guided breast cancer surgery, facilitating intraoperative discrimination between malignant and normal breast tissues [88]. Notably, spatial organization of GLUT1 may provide additional biological information beyond overall expression levels. Phosphorylated GLUT1 enrichment on epithelial cell surfaces was observed specifically in recurrent breast ductal carcinoma in situ, and a machine-learning model incorporating this spatial feature achieved 90% accuracy in identifying recurrent lesions [89]. These observations suggest that spatial and functional features of GLUT1 may provide information beyond bulk expression levels. However, whether such spatially resolved measurements retain predictive value across independent patient cohorts remains uncertain. External multicenter validation, standardized assessment of phosphorylated GLUT1, and reproducible spatial-analysis pipelines will therefore be required before clinical implementation.

## 6. Therapeutic Targeting of GLUT1 in Tumor Cells

Against this background, GLUT1 represents a potential therapeutic target when tumor cells are functionally dependent on GLUT1-mediated glucose uptake. Early studies focused mainly on pharmacological inhibition of glucose transport, whereas subsequent work has extended GLUT1-directed strategies to treatment sensitization, non-metabolic functions, regulated cell death, and tumor-targeted delivery. Representative GLUT1-based therapeutic approaches, experimental models, and stages of development are summarized in Table 1.

Several small-molecule compounds have been used to test whether dependence on GLUT1-mediated glucose uptake can be therapeutically exploited. WZB117 inhibits glucose transport, reduces glycolytic flux and intracellular adenosine triphosphate (ATP) levels, and suppresses tumor growth in preclinical models [90]. STF-31 was initially identified through a chemical synthetic-lethality screen in von Hippel-Lindau (*VHL*)-deficient renal cell carcinoma, in which highly glycolytic tumor cells showed marked dependence on GLUT1-mediated glucose uptake [91]. Subsequent chemical-genetic studies, however, identified nicotinamide phosphoribosyltransferase (NAMPT) as an additional cellular target of STF-31-like compounds, indicating that their antitumor activity cannot be attributed solely to GLUT1 inhibition [92]. BAY-876 has substantially greater selectivity for GLUT1 and can inhibit tumors characterized by high aerobic glycolysis and relatively low oxidative phosphorylation, such as retinoblastoma protein 1 (RB1)-positive triple-negative breast cancer (TNBC) [16,93,94]. Its original pharmacological characterization showed single-digit nanomolar potency and more than 100-fold selectivity over GLUT2, GLUT3, and GLUT4. Together, these observations indicate that sensitivity to GLUT1 inhibition depends more on functional metabolic dependence than on GLUT1 expression alone.

Beyond dedicated GLUT1 inhibitors, several clinically used drugs and naturally derived compounds have been reported to interfere with GLUT1-dependent tumor metabolism through distinct mechanisms. Aspirin, dihydroartemisinin, plumbagin/genistein-based formulations, and the combination of Coptis chinensis with dried ginger primarily reduce GLUT1 expression or protein abundance [95,96,97,98]. By contrast, citalopram directly suppresses GLUT1 transport function without decreasing GLUT1 expression [99]. These agents therefore engage the GLUT1 axis at different regulatory levels rather than constituting a uniform class of GLUT1 inhibitors. Their effects ultimately converge on reduced GLUT1-dependent glucose utilization, either by lowering transporter abundance or by directly impairing transport activity.

GLUT1 can also be therapeutically exploited without suppressing its expression or transport function. Pharmacological ascorbate provides a distinct example. In gastric cancer, high GLUT1 expression is associated with greater sensitivity to pharmacological ascorbate, whereas GLUT1 depletion attenuates this response; pharmacological ascorbate also enhances the efficacy of oxaliplatin in GLUT1-high tumors [100]. This relationship does not imply that ascorbate directly inhibits GLUT1. Rather, mechanistic studies in *KRAS* (KRAS proto-oncogene)- or *BRAF* (B-Raf proto-oncogene)-mutant colorectal cancer have shown that dehydroascorbate (DHA), the oxidized form of vitamin C, is transported through GLUT1. Increased GLUT1-mediated DHA uptake depletes intracellular reducing capacity, promotes oxidative stress, inhibits glyceraldehyde-3-phosphate dehydrogenase (GAPDH), and ultimately disrupts glycolysis and cellular energy homeostasis [101]. In this setting, GLUT1 functions as a determinant of therapeutic susceptibility by facilitating DHA uptake, rather than as a transporter that is itself pharmacologically inhibited.

GLUT1 also influences treatment response through mechanisms that are not confined to substrate transport. At the metabolic level, increased GLUT1-dependent glucose uptake can support adaptation to treatment-induced stress. In laryngeal carcinoma, hypoxia-associated GLUT1 upregulation is accompanied by activation of the PI3K/AKT/mTOR pathway and reduced radiosensitivity []. In lung cancer, however, GLUT1 has also been shown to regulate EGFR through a distinct protein–protein interaction. GLUT1 preferentially associates with activated, phosphorylated EGFR and limits its ubiquitin-mediated degradation, thereby maintaining receptor abundance and downstream signaling [10]. Subsequent work further localized this interaction to the extracellular/transmembrane region of EGFR and showed that its strength depends on EGFR activation status [7]. The GLUT1-EGFR interaction is therefore mechanistically distinct from GLUT1-mediated glucose transport and provides an example of how GLUT1 can influence drug response independently of its canonical transport function.

Recent studies of disulfidptosis have further broadened the therapeutic relevance of GLUT1-dependent metabolism. This form of regulated cell death occurs preferentially in cancer cells with high expression of solute carrier family 7 member 11 (*SLC7A11*), which continuously imports cystine and consequently imposes a high demand for reduced nicotinamide adenine dinucleotide phosphate (NADPH) to maintain intracellular redox balance. When glucose availability is limited or glucose transport is pharmacologically inhibited, NADPH production becomes insufficient to accommodate this cystine burden. The resulting accumulation of intracellular disulfides promotes aberrant disulfide bonding in actin cytoskeletal proteins, followed by filamentous actin contraction, cytoskeletal collapse, and cell death. Importantly, glucose-transporter inhibitors have been shown to induce disulfidptosis in *SLC7A11*-high cancer cells and to suppress the growth of *SLC7A11*-high tumors in vivo [102]. Susceptibility to disulfidptosis is also shaped by upstream regulation of GLUT1. In pancreatic cancer, lncRNA TMEM105 stabilizes β-catenin and enhances c-MYC-dependent *SLC2A1* transcription, thereby increasing GLUT1 expression and glucose utilization [43]. Maintenance of this metabolic program protects tumor cells from disulfide stress, whereas disruption of the lncRNA TMEM105/β-catenin/c-MYC/GLUT1 axis increases their sensitivity to disulfidptosis. This finding links transcriptional control of GLUT1 to a defined form of metabolic cell death and provides a rationale for combining glucose restriction with interventions that increase cystine or disulfide stress. This strategy has already been incorporated into stimulus-responsive nanotherapeutic platforms. A degradable ultrasound-radiation-activated nanomedicine (SPCP/CCP@Bay) combines a degradable sonodynamic pseudo-conjugated polymer (SPCP), a cystine-containing polymer (CCP), and BAY-876 [103]. After tumor accumulation, ultrasound irradiation promotes BAY-876 release, perturbs intracellular redox balance, and increases cystine availability from CCP. Restriction of glucose utilization together with the increased cystine burden amplifies disulfide stress and induces disulfidptosis. The same treatment also promotes immunogenic cell death and enhances the antitumor effect of programmed cell death protein 1 (PD-1) blockade in preclinical bladder cancer models [103].

**Figure 4:**
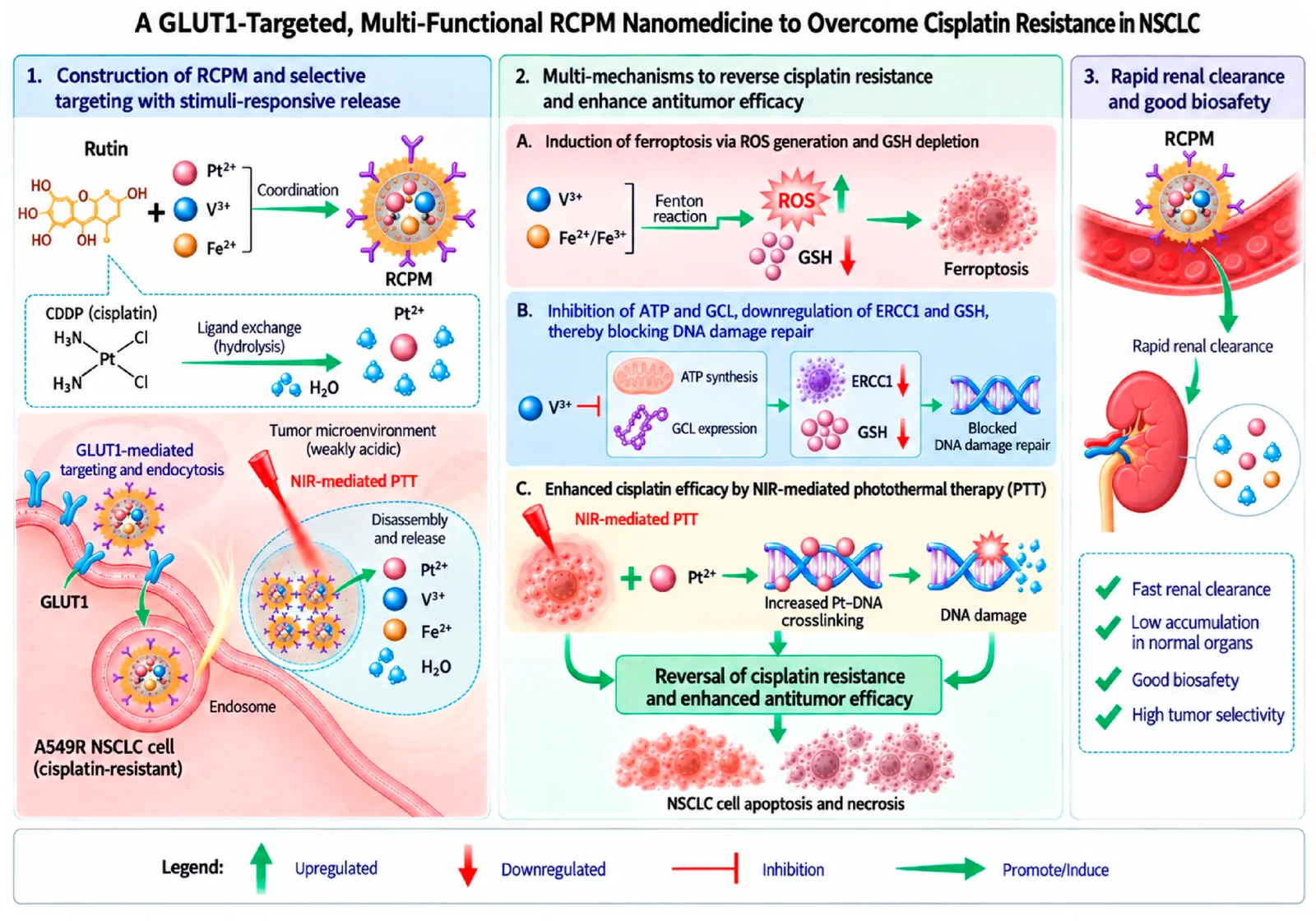
A GLUT1-targeted, multi-functional RCPM nanomedicine to overcome cisplatin resistance in NSCLC. (**1**) Construction, GLUT1-mediated targeting, and stimuli-responsive release of RCPM. A GLUT1-targeted nanodrug (RCPM) is assembled by coordinating the natural polyphenol rutin with Pt-, V-, and Fe-containing components. The Pt-containing component is generated from cisplatin (CDDP) through ligand exchange/aquation. Interaction between rutin and GLUT1 promotes preferential uptake of RCPM by GLUT1-overexpressing cisplatin-resistant A549R cells. Following tumor accumulation, the coordination structure becomes destabilized in the weakly acidic tumor microenvironment and under near-infrared (NIR) irradiation, resulting in release of the active metal-containing components. (**2**) Cooperative mechanisms underlying reversal of cisplatin resistance. V- and Fe-associated reactions increase reactive oxygen species (ROS) production and promote ferroptosis. V^3+^ additionally suppresses adenosine triphosphate (ATP) synthesis and glutamate-cysteine ligase (GCL) expression, leading to reduced excision repair cross-complementation protein 1 (ERCC1) expression and glutathione (GSH) availability. These changes impair DNA repair and diminish GSH-mediated cisplatin detoxification. In parallel, NIR-mediated photothermal therapy (PTT) enhances Pt-DNA crosslinking and DNA damage. Together, these mechanisms restore cisplatin sensitivity and increase tumor-cell killing. (**3**) Renal clearance and biosafety. Following therapeutic action, RCPM and/or its degradable components undergo renal clearance, limiting prolonged retention in normal tissues. Preferential GLUT1-mediated tumor uptake together with subsequent clearance contributes to the favorable biodistribution and biosafety observed in the preclinical model.

Other delivery systems use GLUT1 either as a direct therapeutic target or as part of a broader strategy to exploit tumor metabolic features [104,105,106]. In pancreatic ductal adenocarcinoma, a programmed nanomedicine co-delivering CD71-aptamer-linked GLUT1 small interfering RNA (siRNA) and the nuclear factor-κB (NF-κB) inhibitor TPCA-1 reduced glucose uptake and aerobic glycolysis in tumor cells while limiting metabolic support from activated pancreatic stellate cells. TPCA-1 also restrained compensatory oxidative metabolism under glucose-restricted conditions, resulting in greater tumor control in an orthotopic model [104]. A GLUT1-targeted nanodrug (RCPM) was developed for cisplatin-resistant NSCLC by coordinating the natural polyphenol rutin with Pt-, V-, and Fe-containing components. Interaction between rutin and GLUT1 facilitates preferential uptake by GLUT1-overexpressing A549R cells. After tumor accumulation, the coordination structure becomes destabilized in the weakly acidic TME and under near-infrared (NIR) irradiation, releasing the active components. V- and Fe-associated reactions increase reactive oxygen species (ROS) production and promote ferroptosis. In parallel, V^3+^ suppresses ATP synthesis and glutamate-cysteine ligase (GCL) expression, leading to reduced excision repair cross-complementing protein 1 (ERCC1) expression and GSH availability, thereby weakening DNA repair and cisplatin detoxification. NIR-mediated photothermal therapy (PTT) further enhances Pt-DNA crosslinking and DNA damage. These effects cooperate to restore cisplatin sensitivity and increase tumor-cell killing [106]. Figure 4 summarizes the construction of RCPM, GLUT1-mediated cellular targeting, the principal resistance-reversal mechanisms, and renal clearance.

**Table 1:** Representative GLUT1-directed therapeutic strategies in cancer.

Compound/Approach	Target/Mechanism	Tumor Model	Stage of Development	Main Limitations
WZB117 [90]	Early small-molecule inhibitor of GLUT1-mediated glucose transport; reduces glucose uptake, glycolytic flux, and ATP, leading to cell-cycle arrest and growth inhibition.	NSCLC and breast cancer cell lines; xenograft mouse model.	Preclinical: in vitro/in vivo.	Limited transporter selectivity and inhibition of glucose transport in normal erythrocytes reduce tumor specificity. Evidence remains preclinical; human PK, therapeutic window, and long-term systemic safety are undefined.
STF-31 [91,92]	Initially attributed to GLUT1 inhibition in *VHL*-deficient renal cell carcinoma; subsequent studies identified NAMPT inhibition as a major cytotoxic mechanism.	*VHL*-deficient RCC cell lines; xenograft mouse model.	Preclinical: in vitro/in vivo.	Target ambiguity limits interpretation as selective GLUT1 therapy, and *VHL* dependence may not generalize broadly. Clinically relevant PK, exposure-response relationships, and safety remain undefined.
BAY-876 [16,93]	Potent, reversible, and highly selective GLUT1 inhibitor; suppresses glucose uptake and glycolysis. Sensitivity is greater in tumors with strong glycolytic dependence.	TNBC and HNSCC cell lines; xenograft mouse model.	Preclinical: in vitro/in vivo.	Biochemical selectivity does not ensure tumor selectivity. Response depends on metabolic subtype and may be limited by adaptive bypass; normal-tissue GLUT1 liability, human PK, and long-term safety remain unresolved.
Aspirin [95]	Indirectly lowers GLUT1 expression by suppressing NF-κB/HIF-1α signaling, thereby reducing GLUT1-mediated glucose uptake and HCC proliferation.	HCC cell lines; xenograft mouse model.	Preclinical: in vitro/in vivo.	A pleiotropic drug without GLUT1 selectivity; evidence is largely confined to HCC models. GLUT1-specific target engagement, anticancer dose–response relationships, PK/PD, and long-term safety remain unestablished.
Dihydroartemisinin [96]	Binds YAP1 and disrupts YAP1/HIF-1α signaling, lowering GLUT1 expression and GLUT1-dependent aerobic glycolysis; it also modifies the immune/metabolic tumor milieu.	HCC cell lines; xenograft mouse model.	Preclinical: in vitro/in vivo.	Acts upstream of GLUT1 and is not a selective GLUT1 inhibitor; YAP1 has broad pleiotropic functions. Evidence is centered on HCC; cross-tumor generalizability, tumor-specific PK/PD, and long-term safety remain insufficiently defined.
Plumbagin/genistein nanoliposomal formulation [97]	The combination suppresses PI3K/AKT3 signaling and decreases GLUT1 transporter abundance, with concomitant antiproliferative and antiangiogenic effects.	Prostate cancer cell lines; xenograft mouse model.	Preclinical: in vitro/in vivo.	Multicomponent, multitarget activity makes the GLUT1-specific contribution difficult to isolate. Evidence is limited to prostate cancer models; nanoliposomal manufacturing, component-specific PK, biodistribution, and long-term toxicity lack clinical characterization.
Coptis chinensis + dried ginger [98]	Complex herbal combination that suppresses glucose metabolism and downregulates *SLC2A1* and *LDHA* expression rather than directly inhibiting GLUT1 transport.	Gastric cancer cell lines; xenograft mouse model.	Preclinical: in vitro/in vivo.	Complex composition and polypharmacology limit attribution of efficacy to *SLC2A1*/GLUT1. Extract standardization, active constituents, dose equivalence, and cross-tumor applicability remain uncertain; human oncology PK/PD and long-term safety data are lacking.
Citalopram [99]	Directly binds GLUT1 at the E380 site and suppresses transporter function and glycolytic flux without reducing GLUT1 expression.	HCC cell lines; xenograft mouse model; retrospective HCC patient cohort.	Preclinical: in vitro/in vivo; retrospective clinical evidence.	Anticancer dosing, GLUT1 target engagement, and efficacy have not been prospectively established in patients; retrospective associations do not establish causality. Normal-tissue selectivity, oncology PK/PD, and long-term combination safety require further study.
Pharmacological ascorbate/DHA [100,101]	Does not inhibit GLUT1. High GLUT1 facilitates DHA uptake; intracellular DHA reduction consumes reducing capacity, increases oxidative stress, and can inhibit GAPDH, creating a metabolic liability in susceptible tumors.	Gastric cancer and *KRAS*/*BRAF*-mutant colorectal cancer cell lines; xenograft mouse model.	Preclinical: in vitro/in vivo.	GLUT1 expression alone is insufficient to predict response; genotype and redox state also modify sensitivity. Clinical efficacy in biomarker-selected patients, optimal combinations, PK/PD relationships, and long-term safety remain to be established.
GLUT1 inhibition-induced disulfidptosis [102]	GLUT1 inhibition limits glucose-derived NADPH in *SLC7A11*-high cells, causing disulfide accumulation, aberrant actin disulfide bonding, filamentous actin collapse, and disulfidptosis.	*SLC7A11*-high NSCLC and RCC cell lines; xenograft mouse model.	Preclinical: in vitro/in vivo.	Applicable mainly to *SLC7A11*-high, redox-vulnerable tumors rather than all GLUT1-high cancers. GLUT blockade may affect normal glucose-dependent tissues; the human therapeutic window, PK, and chronic safety remain unknown.
A degradable ultrasound-radiation-activated nanomedicine (SPCP/CCP@Bay) [103]	Ultrasound-triggered release of BAY-876 restricts glucose utilization, while CCP increases the cystine burden; combined redox stress induces disulfidptosis and ICD and can enhance PD-1 blockade.	Bladder cancer cell lines; syngeneic mouse model.	Preclinical: in vitro/in vivo.	Efficacy depends on ultrasound delivery, formulation behavior, and a disulfidptosis-permissive metabolic state; evidence is limited to bladder cancer models. Human acoustic dosing, manufacturing reproducibility, PK, clearance, off-target exposure, and long-term safety remain unresolved.
A programmed nanomedicine co-delivering CD71-aptamer-linked GLUT1 siRNA and NF-κB inhibitor TPCA-1 [104]	Sequential TME/tumor-cell targeting: TPCA-1 suppresses NF-κB-driven stromal support and compensatory OXPHOS, while CD71-directed GLUT1 siRNA silences tumor-cell GLUT1 and reduces glycolysis.	PDAC cell lines/PSC co-culture; xenograft mouse model.	Preclinical: in vitro/in vivo; PK/biodistribution evaluated.	Complex multicomponent delivery and dual-target biology complicate manufacturing and mechanistic attribution. Evidence centers on PDAC with high GLUT1/CD71 expression; stromal heterogeneity, human PK, durability, and long-term safety remain major translational gaps.
A GLUT1-targeted nanodrug (RCPM) [106]	Rutin-based GLUT1 targeting with Pt/V/Fe coordination; acidic TME and NIR irradiation release active components. V^3+^/Fe^2+^ increase ROS generation and promote ferroptosis; V^3+^ reduces ATP/GCL/ERCC1/GSH, while PTT enhances Pt-DNA damage, collectively restoring cisplatin sensitivity.	Cisplatin-resistant NSCLC cell lines; xenograft mouse model.	Preclinical: in vitro/in vivo; biodistribution/clearance evaluated.	Evidence is restricted to cisplatin-resistant NSCLC; component-specific contributions and broader clinical generalizability remain uncertain. Rapid renal clearance and short-term murine safety do not establish human PK, metal disposition, repeated-dose toxicity, or long-term safety.

Abbreviations: GLUT1, glucose transporter 1; ATP, adenosine triphosphate; NSCLC, non-small-cell lung cancer; PK, pharmacokinetics; *VHL*, von Hippel-Lindau; NAMPT, nicotinamide phosphoribosyltransferase; RCC, renal cell carcinoma; TNBC, triple-negative breast cancer; HNSCC, head and neck squamous cell carcinoma; NF-κB, nuclear factor-κB; HIF-1α, hypoxia-inducible factor 1-alpha; HCC, hepatocellular carcinoma; PK/PD, pharmacokinetics/pharmacodynamics; YAP1, Yes-associated protein 1; PI3K, phosphatidylinositol-3-kinase; AKT3, serine/threonine kinase 3; *SLC2A1*, solute carrier family 2 member 1; *LDHA*, lactate dehydrogenase A; DHA, dehydroascorbate; GAPDH, glyceraldehyde-3-phosphate dehydrogenase; *KRAS*, KRAS proto-oncogene, GTPase; *BRAF*, B-Raf proto-oncogene, serine/threonine kinase; NADPH, reduced nicotinamide adenine dinucleotide phosphate; *SLC7A11*, solute carrier family 7 member 11; SPCP, sonodynamic pseudo-conjugated polymer; CCP, cystine-containing polymer; ICD, immunogenic cell death; PD-1, programmed cell death protein 1; CD71, transferrin receptor 1; siRNA, small interfering RNA; TME, tumor microenvironment; OXPHOS, oxidative phosphorylation; PDAC, pancreatic ductal adenocarcinoma; PSCs, pancreatic stellate cells; NIR, near-infrared; ROS, reactive oxygen species; GCL, glutamate cysteine ligase; ERCC1, excision repair cross-complementation protein 1; GSH, glutathione; PTT, photothermal therapy.

Collectively, the broader therapeutic consequences of GLUT1 inhibition are further shaped by its cell-type-specific roles within the TME, where non-malignant cellular compartments can exhibit distinct, and in some cases opposing, requirements for GLUT1-dependent glucose metabolism.

## 7. Therapeutic Targeting of GLUT1 in Non-Malignant Cells of the TME

The therapeutic consequences of GLUT1-dependent metabolism within the TME are strongly determined by the cellular compartment in which this dependency resides. In selected stromal and myeloid populations, GLUT1 supports metabolic states that favor tumor progression or immune suppression, whereas activated antitumor lymphocytes also require glucose uptake to sustain proliferation and effector activity. Thus, targeting GLUT1 in the TME requires consideration not only of transporter expression, but also of which cell population is metabolically dependent on GLUT1 and how that dependency influences neighboring malignant or immune cells.

### 7.1. Tumor-Associated Neutrophils (TANs)

Under homeostatic conditions, neutrophils predominantly rely on intracellular glycogen stores to sustain their metabolic demands. Upon activation, however, they rapidly upregulate surface GLUT1 expression and undergo a marked increase in glycolytic flux. This metabolic transition supports several major neutrophil effector functions [107,108]. Within the TME, TANs comprise a highly heterogeneous and functionally plastic population whose phenotypic states are tightly orchestrated by local environmental cues. These cells can adopt either antitumor or protumor programs, reflecting distinct metabolic and immunological states [109,110].

In murine lung adenocarcinoma, increased GLUT1 expression and glucose metabolism are particularly evident in a tumor-promoting TAN population, which is characterized by high expression of the immunomodulatory molecules sialic acid binding Ig-like lectin 5 (SiglecF) and PD-L1. Selective deletion of GLUT1 in neutrophils alters intratumoral neutrophil turnover and shifts the TAN compartment away from this tumor-supportive state, resulting in reduced tumor growth and enhanced radiosensitivity [111]. These findings provide direct genetic evidence that neutrophil-intrinsic GLUT1 metabolism can influence both TAN composition and therapeutic response. However, whether pharmacological GLUT1 inhibition can reproduce this compartment-restricted effect remains to be established.

### 7.2. Tumor-Associated Fibroblasts (CAFs)

Several studies suggest that GLUT1 upregulation accompanies the acquisition of activated, proinflammatory, or profibrotic phenotypes in fibroblasts [112,113,114]. Within tumors, GLUT1-related CAF–tumor crosstalk can operate in more than one direction. In ovarian cancer, CAF-intrinsic GLUT1 increases stromal glycolysis and lactate production, and the resulting metabolic output acts on adjacent tumor cells to promote proliferative and invasive signaling [115].

CAFs can also regulate GLUT1-dependent metabolism without being the cellular site of GLUT1 activation. In hepatocellular carcinoma, CAF-derived ANGPTL4 reprograms GLUT1-dependent glycolysis in neighboring malignant cells, increasing lactate production and promoting lactate-dependent epigenetic regulation of tumor cell *PD-L1* expression, with consequent suppression of CD8^+^ T cell activity [76]. Thus, CAFs can influence antitumor immunity indirectly by imposing a GLUT1-dependent metabolic state on malignant cells, whereas in other settings their own GLUT1-dependent glycolysis constitutes the relevant stromal dependency.

Accordingly, pharmacological or genetic inhibition of GLUT1, or disruption of its upstream regulators, protein kinase M zeta isoform (PKMζ) and Src (encoded by the well-known proto-oncogene tyrosine-protein kinase *Src*), attenuates CAF activation and tumor-promoting activity [113,114]. These observations suggest two related therapeutic entry points: suppression of CAF-intrinsic glucose metabolism and disruption of CAF-driven metabolic reprogramming of malignant cells. A CAF-derived extracellular-vesicle formulation of BAY-876 has been shown to reverse activated stromal features, reduce lactate accumulation and matrix stiffness, and increase intratumoral CD8^+^ T cell infiltration [116]. By contrast, biomimetic nanoparticles incorporating hybrid cancer cell–CAF membranes target both stromal and malignant metabolic compartments [6]. Together, these approaches illustrate that the therapeutic value of GLUT1-directed intervention may derive either from selective stromal reprogramming or from coordinated disruption of tumor–stroma metabolic coupling.

### 7.3. Tumor-Associated Macrophages (TAMs)

GLUT1-related macrophage regulation likewise encompasses both tumor-cell-driven and macrophage-intrinsic metabolic programs. In lung adenocarcinoma, tumor-cell CD155 enhances YAP/TEAD1-dependent GLUT1 expression and glycolysis, increasing lactate production and thereby promoting an immunosuppressive macrophage phenotype [117]. In this configuration, GLUT1 is primarily engaged in malignant cells, whereas macrophages represent the responding immune compartment.

By contrast, several studies identify GLUT1 itself as a macrophage-intrinsic dependency. In lung squamous cell carcinoma, *SLC2A1* is enriched in SPP1^+^ (secreted phosphoprotein 1) macrophages, and inhibition of GLUT1 attenuates hypoxia-associated macrophage polarization [118]. More direct evidence comes from NSCLC, where TAM-specific *SLC2A1* deletion increases the spatial homogeneity and effector function of intratumoral CD8^+^ T cells and improves sensitivity to anti-PD-L1 therapy, whereas tumor cell-specific *SLC2A1* depletion does not reproduce this phenotype [71]. Importantly, this immune restoration is not accompanied by increased glucose uptake by CD8^+^ T cells, indicating that macrophage GLUT1 regulates T cell function through mechanisms that extend beyond simple competition for extracellular glucose.

A related macrophage-intrinsic dependency occurs in glioblastoma, where tumor-derived signals induce GLUT1-dependent glycolysis in monocyte-derived macrophages and establish a lactate-associated immunosuppressive transcriptional program that inhibits T cell activity [75]. Taken together, these studies distinguish two biologically different forms of GLUT1-mediated macrophage regulation: tumor-cell GLUT1 can alter macrophage state through extracellular metabolic signals, whereas macrophage-intrinsic GLUT1 can directly sustain immunosuppressive myeloid functions. This distinction is important for therapeutic design because the appropriate cellular target differs between the two settings.

Current treatment strategies exploit both configurations. TAM-specific genetic deletion provides proof of principle that direct interruption of macrophage *SLC2A1* can enhance checkpoint blockade [71]. In contrast, the Gel@B-B strategy, an injectable thermogel loaded with the GLUT1 inhibitor BAY-876 and the PD-1/PD-L1 blocker BMS-1, primarily inhibits tumor-cell GLUT1-dependent lactate production with BAY-876, thereby reducing the accumulation of lactate-associated TAMs and regulatory T (Treg) cells, while concomitant PD-1/PD-L1 blockade counteracts adaptive immune resistance [119]. Thus, the immunological benefit of GLUT1 inhibition in this model arises largely from modifying the metabolic signals received by non-malignant immune cells rather than from demonstrating macrophage-selective pharmacological inhibition.

### 7.4. T Cell Dependence on GLUT1

The therapeutic interpretation of GLUT1 becomes more complex in lymphocytes because GLUT1 can support, rather than suppress, antitumor immunity. GLUT1 expression is relatively low in quiescent T cells but increases following T cell receptor activation to accommodate the bioenergetic and biosynthetic requirements of clonal expansion and effector differentiation. Genetic loss of GLUT1 directly impairs glucose uptake, growth, proliferation, and effector differentiation in activated CD4^+^ T cells [120], establishing a cell-intrinsic requirement for GLUT1 during effector T cell activation.

The role of GLUT1 in Treg cells is more complex and context-dependent. Toll-like receptor (TLR) signaling activates the PI3K/Akt/mTORC1 pathway to upregulate GLUT1 expression and promote Treg proliferation, albeit at the expense of immunosuppressive capacity. In contrast, the lineage-defining transcription factor Forkhead box P3 (FOXP3) antagonizes this signaling axis, restrains glycolysis, and favors oxidative metabolism, thereby preserving the immunosuppressive function and lineage stability of Treg cells [121]. These divergent metabolic requirements indicate that the consequences of GLUT1 modulation cannot be generalized across T cell subsets. Similarly, GLUT1-mediated glycolytic reprogramming supports the differentiation of inflammatory T helper 17 (Th17) cells, whereas inhibition of GLUT1 attenuates IL-17 production and impairs Th17 effector function [122].

CD8^+^ T cells also require substantial metabolic support during proliferation and acquisition of effector function [123]. This dependence can itself be therapeutically exploited: enforced GLUT1 expression in CAR-T cells increases glucose consumption and metabolic capacity and improves persistence and antitumor activity in preclinical models [124,125].

Within tumors, however, this lymphocyte-intrinsic requirement for glucose is opposed by GLUT1-dependent metabolism in malignant cells. Tumor cell stabilization of GLUT1 can increase glucose consumption and restrict the metabolic fitness of CD8^+^ T cells [70], while lactate-rich conditions further impair antitumor T cell metabolism [126]. The therapeutic implication is therefore bidirectional: GLUT1 activity may need to be suppressed in malignant or immunosuppressive stromal/myeloid compartments, yet preserved or even augmented in effector lymphocytes.

This principle is reflected in emerging metabolic-immunotherapeutic strategies. Nanovesicle and photodynamic platforms incorporating GLUT1 inhibitors are designed primarily to restrict tumor cell glucose metabolism and thereby reduce metabolic immune suppression, while simultaneously activating innate or adaptive immunity [127,128]. In these settings, improved CD8^+^ T cell infiltration and activity are secondary to metabolic intervention in the tumor compartment rather than direct inhibition of T cell GLUT1.

Taken together, the therapeutic effects of GLUT1 modulation within the TME are defined by the cellular origin and direction of the metabolic interaction. GLUT1-dependent programs in TANs, CAFs, and TAMs can sustain tumor-promoting or immunosuppressive states, whereas activated antitumor lymphocytes retain a substantial requirement for glucose transport. This opposing biology makes cell-compartment selectivity a central constraint on GLUT1-directed therapy.

## 8. Discussion

### 8.1. Current Understanding and Unresolved Challenges

The evidence reviewed here positions GLUT1 not as a uniform marker of aerobic glycolysis, but as a context-dependent metabolic dependency whose biological and clinical significance varies across tumor lineages, disease states, and cellular compartments. High *SLC2A1* expression does not invariably indicate greater glycolytic dependence, adverse prognosis, or sensitivity to GLUT1 inhibition. This distinction provides a coherent basis for interpreting many of the apparently discordant observations discussed throughout this review and argues against using GLUT1 abundance as an isolated biological or therapeutic readout.

A major unresolved question is how GLUT1 dependence is established, maintained, and remodeled over time. Although transcriptional, post-transcriptional, and post-translational regulators have been identified, their relative hierarchy and temporal coordination during tumor progression or treatment remain poorly defined. Elevated GLUT1 may therefore reflect an established metabolic dependency, a stress-induced adaptive response, or a state that is subsequently reconfigured under therapeutic pressure. This uncertainty is also relevant to the conflicting prognostic associations reported across tumor types, because similar levels of GLUT1 expression may correspond to substantially different metabolic states.

The therapeutic window represents a second and more immediate challenge. GLUT1-dependent metabolism can sustain tumor-promoting functions in malignant, stromal, and myeloid compartments, whereas activated antitumor lymphocytes and physiologically vulnerable tissues such as the BBB also retain important requirements for GLUT1-mediated glucose transport. Therapeutic selectivity therefore cannot be inferred from molecular selectivity for GLUT1 alone. It also depends on the magnitude and duration of inhibition, systemic exposure, and, critically, the cellular and tissue distribution of target engagement. Cell-restricted genetic models provide proof of principle that selective disruption of GLUT1 in defined disease-promoting compartments can be beneficial, whereas current pharmacological inhibitors and delivery systems achieve variable degrees of such compartmental selectivity.

Metabolic plasticity further constrains both treatment durability and biomarker performance. Tumor cells may preserve metabolic fitness by increasing reliance on alternative glucose transporters such as GLUT3, shifting toward oxidative metabolism, or engaging other stress-adaptation programs. As these compensatory states emerge, GLUT1 expression may become progressively uncoupled from therapeutic sensitivity. Consistent with this complexity, candidate biomarkers identified to date—including RB1 status, GLUT1 membrane or spatial localization, and *SLC7A11*-associated metabolic vulnerability—remain context specific rather than broadly predictive across malignancies.

Finally, the most advanced GLUT1-directed therapeutic platforms remain predominantly preclinical. Approaches such as SPCP/CCP@Bay, RCPM, Gel@B-B, CAF-derived extracellular-vesicle formulations, and related metabolic–immunotherapeutic nanoplatforms illustrate the feasibility of combining GLUT1 modulation with regulated cell death, redox perturbation, local immune remodeling, or reversal of drug resistance. Their clinical relevance, however, cannot be inferred from antitumor efficacy alone. Human pharmacokinetics, biodistribution, long-term biocompatibility, organ clearance, manufacturing reproducibility, and the contribution of individual platform components to both efficacy and toxicity remain insufficiently characterized.

### 8.2. Future Directions for Clinical Translation

Future development should first establish functional GLUT1 dependence prospectively rather than infer it from *SLC2A1* expression alone. Conventional pathological assessment should be integrated with GLUT1 membrane localization, glucose flux, mitochondrial capacity, compensatory transporter activity, and spatial features of the TME. Complementary molecular markers should also be incorporated where biologically justified. For example, RB1 status, compensatory GLUT3 expression, or *SLC7A11*-associated metabolic vulnerability may refine patient selection in selected tumor contexts. The immediate objective should not be to construct a universal biomarker panel, but to determine which combinations improve the sensitivity and specificity for identifying functionally GLUT1-dependent tumors within defined cancer types. Imaging- and machine-learning-based classifiers should be evaluated according to the same principle and validated in independent, multicenter cohorts using standardized acquisition and analytical procedures.

The temporal evolution of GLUT1 dependence will be equally important. Longitudinal profiling before, during, and after treatment could define how a pre-existing GLUT1-dependent state changes under therapeutic pressure and identify the timing, persistence, and reversibility of compensatory metabolic programs. Such information could distinguish early adaptive responses from more durable metabolic rewiring and provide a stronger mechanistic basis for treatment sequencing. For tumors that increase GLUT3-mediated glucose uptake, interference with alternative transport may be relevant; for tumors that shift toward oxidative metabolism, mitochondrial or oxidative-pathway inhibition may be more appropriate; and in *SLC7A11*-high tumors, glucose restriction may be exploited to increase susceptibility to disulfide stress. Combination partners, dose intensity, and treatment schedules should therefore integrate the adaptive metabolic state induced by GLUT1 inhibition with tumor-specific pharmacological and safety constraints, with the aim of increasing the depth and durability of response without imposing unnecessary metabolic toxicity.

The therapeutic window should also be defined experimentally at the level of cellular target engagement rather than bulk tumor exposure. Future studies should quantify pharmacodynamic inhibition separately in malignant cells, disease-promoting stromal and myeloid populations, effector lymphocytes, and physiologically vulnerable normal tissues. This distinction is essential because the desired direction of GLUT1 modulation differs among compartments. Local administration, cell-biased delivery, ligand-guided targeting, and microenvironment-responsive release therefore represent complementary approaches to increasing therapeutic selectivity. Existing thermogel, extracellular-vesicle, and stimulus-responsive nanomedicine platforms provide useful proof of concept, but their cellular distribution and on-target pharmacodynamic effects should be measured directly rather than inferred from overall tumor accumulation.

Clinical development of GLUT1-directed nanomedicine and combination therapies will likewise require a more stringent translational framework. Human pharmacokinetics, organ-specific exposure, neurological safety, renal and hepatic clearance, immunogenicity, manufacturing consistency, and long-term toxicity should be incorporated early in development. For multicomponent platforms, the contribution of individual components to efficacy and toxicity should be resolved wherever possible. Pharmacodynamic studies should also establish whether therapeutic benefit derives primarily from target engagement in malignant cells, remodeling of stromal or immune compartments, or both, because these mechanisms imply different safety requirements and different routes to clinical optimization.

A clinically useful GLUT1-directed strategy is therefore unlikely to be defined simply by the availability of a potent inhibitor. More relevant is the identification of a defined state of GLUT1 dependence with a known cellular location, a measurable pharmacodynamic response, and an adaptive escape program that can be anticipated or controlled. Establishing these features prospectively would provide a more rigorous basis for patient selection, combination design, dose optimization, and ultimately the clinical translation of GLUT1-directed precision metabolic therapy.

## Data Availability

No new data were created or analyzed in this study. Data sharing is not applicable to this article.

## Abbreviations

The following abbreviations are used in this manuscript:

*SLC2A1*	Solute carrier family 2 member 1
GLUT1	Glucose transporter 1
BBB	Blood–brain barrier
CNS	Central nervous system
Glut1DS	GLUT1 deficiency syndrome
TME	Tumor microenvironment
HIF-1α	Hypoxia-inducible factor-1alpha
AgNPs	Silver nanoparticles
HRE	Hypoxia-responsive element
SOX2	Sex-determining region Y-box 2
YAP1	Yes-associated protein 1
TEAD1	TEA domain transcription factor 1
CDK7	Cyclin-dependent kinase 7
Zic	Zinc finger of the cerebellum proteins
JMJD2B	Jumonji domain-containing protein 2B
H3K9me3	Histone H3 lysine 9 trimethylation
DLAT	Dihydrolipoyl transacetylase
SALL4	Spalt-like transcription factor 4
H3K18ac	Histone H3 lysine 18 acetylation
lncRNAs	Long non-coding RNAs
METTL3	Methyltransferase-like 3
m6A	N6-methyladenosine
3′ UTR	3′ untranslated region
ceRNAs	Competing endogenous RNAs
TRIM38	Tripartite motif-containing protein 38
CMTM6	CKLF-like MARVEL transmembrane domain-containing 6
*DERL3*	Hypermethylation-induced silencing of derlin-3
USP6NL	Ubiquitin-specific protease 6 N-terminal-like protein
EGFR	Epidermal growth factor receptor
AKT	Serine/threonine kinase
TBK1	TANK-binding kinase 1
mTORC1	Mechanistic target of rapamycin complex 1
DHHC9	DHHC-type palmitoyltransferase 9
PI3K	Phosphatidylinositol-3-kinase
MIOS	Meiosis regulator for oocyte development
KSHV	Kaposi sarcoma-associated herpesvirus
IKKβ	Inhibitor of nuclear factor kappa-B kinase subunit beta
PON2	Paraoxonase 2
STOM	Stomatin
FOXD1	Forkhead box D1
RISC	RNA-induced silencing complex
USP14	Ubiquitin-specific protease 14
NSCLC	Non-small-cell lung cancer
LDHA	Lactate dehydrogenase A
NK	Natural killer cells
NFAT	Nuclear factor of activated T cells
PERK	Protein kinase R-like endoplasmic reticulum kinase
ATF4	Transcription factor 4
CAFs	Tumor-associated fibroblasts
ANGPTL4	Angiopoietin-like 4
H3K18la	Histone H3 lysine 18 lactylation
*PD-L1*	Programmed cell death 1 ligand 1
JAK1	Janus kinase 1
STAT3	Signal transducer and activator of transcription 3
*TRPS1*	Transcriptional repressor GATA binding 1
*ETV1*	ETS variant transcription factor 1
CCL2	C-C motif chemokine ligand 2
ATP	Adenosine triphosphate
*VHL*	Von Hippel-Lindau
NAMPT	Nicotinamide phosphoribosyltransferase
RB1	Retinoblastoma protein 1
TNBC	Triple-negative breast cancer
*KRAS*	KRAS proto-oncogene
*BRAF*	B-Raf proto-oncogene
DHA	Dehydroascorbate
GAPDH	Glyceraldehyde-3-phosphate dehydrogenase
*SLC7A11*	Solute carrier family 7 member 11
NADPH	Nicotinamide adenine dinucleotide phosphate
SPCP/CCP@Bay	Degradable ultrasound radiation-activated nanomedicine
SPCP	Sonodynamic pseudo-conjugated polymer
CCP	Cystine-containing polymer
PD-1	Programmed cell death protein 1
siRNA	Small interfering RNA
NF-κB	Nuclear factor-κB
RCPM	GLUT1-targeted nanodrug
NIR	Near-infrared
ROS	Reactive oxygen species
GCL	Glutamate-cysteine ligase
ERCC1	Excision repair cross-complementation protein 1
PTT	Photothermal therapy
TANs	Tumor-associated neutrophils
SiglecF	Sialic acid binding Ig-like lectin 5
PKMζ	Protein kinase M zeta isoform
TAMs	Tumor-associated macrophages
SPP1	Secreted phosphoprotein 1
Gel@B-B	Injectable thermogel loaded with a GLUT1 inhibitor, BAY-876, and a PD-1/PD-L1 blocker BMS-1
Treg	Regulatory T cells
TLR	Toll-like receptor
FOXP3	Forkhead box P3
Th17	T helper 17
